# FEV_1_ and FVC and systemic inflammation in a spinal cord injury cohort

**DOI:** 10.1186/s12890-017-0459-6

**Published:** 2017-08-15

**Authors:** Jaime E. Hart, Rebekah Goldstein, Palak Walia, Merilee Teylan, Antonio Lazzari, Carlos G. Tun, Eric Garshick

**Affiliations:** 1 Channing Division of Network Medicine, Department of Medicine, Brigham and Women’s Hospital, 401 Park Drive, Landmark Center 3rd Floor West, Boston, MA 02215 USA; 2 Department of Environmental Health, Harvard T.H. Chan School of Public Health, 401 Park Drive, Landmark Center 3rd Floor West, Boston, MA 02215 USA; 3 Harvard Medical School, Boston, 02115 MA USA; 4 Research and Development Service, VA Boston Healthcare System, Boston, MA USA; 5 Divison of Primary Care and Rheumatology Section, VA Boston Healthcare System, Boston University School of Medicine, Boston, MA USA; 6 Department of Physical Medicine and Rehabilitation, VA Boston Healthcare System, Boston, MA USA; 7 Pulmonary, Allergy, Sleep, and Critical Care Medicine Section, Medical Service, VA Boston Healthcare System, Boston, MA USA; 8 VA Boston Healthcare System- West Roxbury Campus, 1400 VFW Parkway, Building 88, Room 18, West Roxbury, MA 02132 USA; 9 VA Boston Healthcare System- Jamaica Plain Campus, 151C- SCI Research, 150 South Huntington Avenue, Jamaica Plain, MA 02130 USA; 10 Department of Epidemiology, University of Washington, 4311 11th Avenue NE, Suite 300, Seattle, WA 98105 USA; 11 Primary Care Clinic, 2nd Floor, Rm, F2-10, 150 South Huntington Avenue, Jamaica Plain, MA 02130 USA

**Keywords:** CRP, Il-6, Systemic inflammation, Pulmonary function, Chronic spinal cord injury

## Abstract

**Background:**

Systemic inflammation has been associated with reduced pulmonary function in individuals with and without chronic medical conditions. Individuals with chronic spinal cord injury (SCI) have clinical characteristics that promote systemic inflammation and also have reduced pulmonary function. We sought to assess the associations between biomarkers of systemic inflammation with pulmonary function in a chronic SCI cohort, adjusting for other potential confounding factors.

**Methods:**

Participants (*n* = 311) provided a blood sample, completed a respiratory health questionnaire, and underwent spirometry. Linear regression methods were used to assess cross-sectional associations between plasma C-reactive protein (CRP) and interleukin-6 (IL-6) with forced expiratory volume in one second (FEV_1_), forced vital capacity (FVC), and FEV_1_/FVC.

**Results:**

There were statistically significant inverse relationships between plasma CRP and IL-6 assessed in quartiles or continuously with FEV_1_ and FVC. In fully adjusted models, each interquartile range (5.91 mg/L) increase in CRP was associated with a significant decrease in FEV_1_ (−55.85 ml; 95% CI: -89.21, −22.49) and decrease in FVC (−65.50 ml; 95% CI: -106.61, −24.60). There were similar significant findings for IL-6. There were no statistically significant associations observed with FEV_1_/FVC.

**Conclusion:**

Plasma CRP and IL-6 in individuals with chronic SCI are inversely associated with FEV_1_ and FVC, independent of SCI level and severity of injury, BMI, and other covariates. This finding suggests that systemic inflammation associated with chronic SCI may contribute to reduced pulmonary function.

**Electronic supplementary material:**

The online version of this article (doi:10.1186/s12890-017-0459-6) contains supplementary material, which is available to authorized users.

## Background

A growing literature supports inverse associations between levels of inflammatory markers and reductions in measures of pulmonary function in both the general population and among populations with chronic disease (e.g. chronic obstructive pulmonary disease (COPD), asthma, end-stage-renal disease) [1–17]. The majority of these studies have been cross-sectional; however, these findings have also been reported in some, but not all, longitudinal studies [18–24]. The consistency of observations across study populations suggests that systemic inflammation, and not pulmonary inflammation alone, may play a role in reductions in pulmonary function.

SCI is a chronic medical condition that is associated with a number of clinical characteristics that promote systemic inflammation, including increases in central fat, decreased mobility due to muscle paralysis, and recurrent infection mainly associated with skin ulcers and urinary tract infections [25–32]. In two reports (*n* = 59 and *n* = 137 persons with chronic SCI) from an SCI cohort with data collected between 2003 and 2007 [13, 14], we observed inverse associations between systemic inflammatory biomarkers and forced expiratory volume in one second (FEV_1_) and forced vital capacity (FVC), but not the FEV1/FVC ratio. Due to the smaller sample sizes of our previous studies, we were unable to adjust for large numbers of potential confounders simultaneously and lacked information on others, leaving concerns regarding residual confounding. Our objective was to determine if these findings were generalizable to another SCI cohort with a larger sample size and information on additional potential confounders.

## Methods

### Study population

As part of ongoing work to identify predictors of adverse health outcomes among individuals with SCI, 360 individuals with chronic SCI were recruited between 8/2009 and 4/2015. Participants were recruited from persons receiving care at VA Boston, from the greater Boston area through advertisement, and by direct mail to persons who had received care at Spaulding Rehabilitation Hospital or Boston University Medical Center, members of the National Spinal Cord Injury Association, and subscribers to New Mobility Magazine. Individuals were eligible if they were 22 years of age or older, were 1 or more years post-injury, had no other neuromuscular disease, did not have a tracheostomy, and were able to breathe without chronic ventilatory support. Participants provided informed consent at the beginning of the study and the study protocol was approved by the Institutional Review Board at VA Boston.

### Outcome assessment

Spirometry was based on the 1994 American Thoracic Society (ATS) standards [33] modified for use in SCI as described previously [34]. After demonstrating the maneuver, participants were instructed to exhale maximally and sustain the effort for at least 6 s. Efforts were made to obtain at least three acceptable efforts from each participant. Short expiratory efforts (less than 6 s) and excessive back extrapolation are common in SCI, but we have demonstrated that FVC and FEV_1_ are reproducible in this population [34]. Therefore, we accepted excessive back extrapolation and efforts less than six seconds if the effort was maximal with an acceptable flow-volume loop, and at least a 0.5-s plateau at RV. The highest values of FEV_1_ and FVC from acceptable efforts were used. Percent-predicted FEV_1_ and FVC were calculated based on NHANES equations [35, 36].

### Inflammatory biomarkers

Blood samples were collected using an ethyle-diamine-tetraacetic acid tube and were processed and stored on the same day as collection. Samples were stored at −80 °C until ready for analysis. High-sensitivity plasma CRP was determined by an immunoturbidimetric assay and IL-6 was determined by an ultra-sensitive enzyme linked immunosorbent assay at the Clinical and Epidemiologic Research Laboratory, Children’s Hospital, Boston.

### Potential confounders

Information was collected on a number of *a priori* potential confounders, including age, race, sex, height, body mass index (BMI), cigarette smoking status (current, former, never) and pack-years of smoking, marijuana smoking status (current, former, never), SCI duration, level, and severity, current use of statins and pulmonary medications (inhaled or oral steroids, long-acting bronchodilators, and short-activing bronchodilators), doctor diagnosed chronic obstructive pulmonary disease (COPD) or asthma, history of chest operation or chest injury, and mobility mode (motorized wheelchair, wheelchair, use of cane/walker, or able to walk unassisted). BMI was calculated from measured height and weight for each participant (self-reported weight was used for 4 participants and self-reported height was used for 28 participants).

SCI level and severity was assessed by exam and medical record review. Motor level and completeness of injury was categorized according to the American Spinal Injury Association Impairment Scale (AIS) [37]. Participants were classified as motor complete, (i.e. no motor function below the neurological level (AIS A or B)); AIS C (i.e. motor incomplete, motor function preserved below the neurological level, and more than half the key muscles below the neurological level not strong enough to overcome gravity); or AIS D (i.e. motor incomplete, motor function preserved below the neurological level, and half or more of key muscles below the neurological level strong enough to overcome gravity). Participants were grouped into cervical motor complete and cervical AIS C, high-thoracic (T1-T6) motor complete (AIS A or B) and AIS C, others with T7 or below motor complete (AIS A or B) and AIS C, and all others (AIS D’s)*.*

### Statistical analyses

Participants were excluded from the current analyses if their SCI level could not be determined (*n* = 3), they had a history of prior stroke (*n* = 2), did not provide a blood sample (*n* = 6) or sufficient blood sample for biomarker assessment (*n* = 7), they did not perform spirometry or did not have an acceptable spirometry effort (*n* = 27), or they had a previous lung resection (*n* = 4), leaving a final analytical sample of 311 participants. There were 62 participants who were recruited from our previous SCI cohort (2003–2007) [14] who were retested. General linear models (PROC GLM, SAS 9.4; SAS Institute Inc., Cary, NC) were used to calculate the average (and 95% confidence interval) FEV1, FVC, or FEV1/FEV ratio within each quartile of CRP or IL-6. The significance of the trends across quartiles was assessed using the median value of each inflammatory biomarker within each quartile. Betas and 95% confidence intervals were calculated for an interquartile range (IQR) increase in each biomarker (PROC GLM, SAS 9.4; SAS Institute Inc., Cary, NC). Basic models included adjustment for age, sex, race, and height (FEV_1_ and FVC models only). Each potential confounder (or group of confounders) was added to the basic model. Fully adjusted models included all *a priori* potential confounders, and parsimonious adjusted models included all potential confounders that were associated with the outcome and exposure when added to the basic models. In sensitivity analyses, we adjusted our final models for laboratory batch to assess potential differences in the biomarker measures over time.

## Results

Participants were mostly male (84%) and white (91%) with a mean age of 54 years, had a wide range of injury levels, with a median injury duration of 14.1 years (Table 1). Most were current or former cigarette smokers with a median of 18 pack-years and were overweight. The majority of participants (90%) had at least two or three acceptable efforts with values of FEV_1_ or FVC within 200 mL and an additional 7% (*n* = 21) had reproducible values of either FEV_1_ or FVC. In 3% (*n* = 9), participants had only one acceptable effort.

**Table 1:** Characteristics of 311 individuals included in the analyses

Characteristic	Mean ± SD or Median (25th percentile – 75th percentile)
Age (yrs)	54.0 ± 14.1
Body mass index (kg/m^2^)	26.9 (22.8–31.2)
Injury duration (yrs)	14.1 (5.3–25.4)
Pack years of smoking^a^	18.0 (5.0–37.2)
CRP (mg/L)	2.4 (1.0–6.9)
IL-6 (pg/mL)	2.1 (1.3–4.4)
FEV_1_ (L)	2.8 ± 0.9
% predicted FEV_1_	77.6 ± 20.9
FVC (L)	3.6 ± 1.1
% predicted FVC	78.3 20.1
FEV1/FVC	0.77 ± 0.11
Characteristic	N (%)
Males	260 (83.6)
Race	
White	282 (90.7)
African American	23 (7.4)
Asian	3 (1.0)
American Indian/Alaskan Native	3 (1.0)
Level of injury	
Motor complete cervical & AIS C	76 (24.4)
Motor complete high thoracic & AIS C	42 (13.5)
Motor complete low thoracic & AIS C	61 (19.6)
All AIS D	132 (42.4)
Mobility mode	
Motorized wheelchair	60 (19.3)
Wheelchair	135 (43.4)
Walk with cane/walker	54 (17.4)
Walk unassisted	62 (19.9)
Cigarette smoking status	
Current	52 (16.7)
Former	132 (42.4)
Never	127 (40.8)
Marijuana smoking status	
Current	37 (11.9)
Former	38 (12.2)
Never	236 (75.9)
Current statin use	97 (31.2)
Any pulmonary medication use	19 (6.1)
Short-acting bronchodilator within 6 h	3 (1.0)
Long-acting bronchodilator within 24 h	13 (4.2)
Current inhaled/oral steroid use	16 (5.1)
Doctor diagnosed COPD or asthma	30 (9.7)
History of chest operation or injury	90 (28.9)

^a^Among current and former smokers only (*N* = 184)

The associations of CRP and IL-6 with FEV_1_ are presented in Table 2. In basic models, increases in either inflammatory biomarker were associated with statistically significant reductions in FEV1. Although all adjusted models were attenuated relative to the basic model (Additional file 1: Table S1), the associations were robust to adjustment for potential confounders. In fully adjusted continuous models, each IQR increase in CRP (5.91 mg/L) was associated with a 55.85 (95% CI:-89.21, −22.49, *p*-value = 0.0012) mL decrease in FEV1, and each IQR increase in IL-6 (3.18 pg/mL) was associated with a 61.39 (95% CI: -115.47, −7.30, *p*-value = 0.027) mL decrease. Associations were similar in parsimonious adjusted models that included only age, sex, race, height, cigarette smoking status and pack-years, marijuana smoking status, doctor diagnosed COPD or asthma, level of injury and mobility mode (dichotomized into wheelchair vs walking aided or unaided), and use of inhaled steroids or long-acting bronchodilators.

**Table 2:** Adjusted mean levels of FEV1 by quartile of inflammatory biomarkers and associations per IQR change

CRP (mg/L)							
	Q1 (0.07–0.99)	Q2 (1.00–2.41)	Q3 (2.42–6.91)	Q4 (6.92–161.56)	p-for trend	β (95% CI) mL FEV1 per 5.91 mg/L CRP	*p*-value
N	77	78	78	78	311	311	311
Basic^a^	3.05 (2.89, 3.22)	2.84 (2.68, 3.00)	2.59 (2.43, 2.75)	2.55 (2.39, 2.71)	0.0002	−63.71 (−99.27,-28.15)	0.0005
Fully adjusted	2.93 (2.76, 3.10)	2.82 (2.67, 2.97)	2.66 (2.51, 2.82)	2.62 (2.46, 2.78)	0.0346	−55.85 (−89.21,-22.49)	0.0012
Parsimonious adjusted^b^	2.93 (2.77, 3.09)	2.81 (2.66, 2.96)	2.67 (2.52, 2.82)	2.62 (2.47, 2.77)	0.0183	−51.83 (−83.92,-19.74)	0.0017
IL-6 (pg/mL)							
	Q1 (0.30–1.26)	Q2 (1.27–2.12)	Q3 (2.13–4.44)	Q4 (4.45–46.8)	p-for trend	β (95% CI) mL FEV1 per 3.18 pg/mL IL-6	*p*-value
N	77	83	76	75	311	311	311
Basic^a^	2.98 (2.81, 3.15)	2.82 (2.66, 2.98)	2.71 (2.54, 2.88)	2.51 (2.34, 2.68)	0.0003	−101.28 (−156.74,-45.83)	0.0004
Fully adjusted	2.90 (2.74, 3.07)	2.75 (2.60, 2.90)	2.77 (2.61, 2.92)	2.61 (2.44, 2.77)	0.0388	−61.39 (−115.47,-7.30)	0.027
Parsimonious adjusted^b^	2.90 (2.75, 3.06)	2.75 (2.60, 2.89)	2.77 (2.61, 2.92)	2.61 (2.45, 2.76)	0.0240	−61.48 (−112.64,-10.33)	0.0192

^a^Adjusted for age, sex, race, and height

^b^Adjusted for age, sex, race, height, smoking status and pack-years, marijuana smoking status, COPD or asthma, current use of steroids and long-acting bronchodilators, level/severity of injury (LOI), and wheelchair use

Similar results were observed in models examining the associations of the inflammatory biomarkers with FVC (Table 3), although the models were slightly more sensitive to adjustment for potential confounders, especially mobility mode (Additional file 2: Table S2). In fully adjusted models, each IQR increase in CRP was associated with a 65.50 (95% CI: -106.61, −24.60, *p*-value = 0.0019) mL decrease in FVC and each IQR increase in IL-6 was associated with a 77.69 (95% CI: -143.93, −11.46, *p*-value = 0.0222) ml decrease. Adjustment for level of injury and mobility mode led to the largest attenuations in the effect estimates. Increases in CRP and IL-6 were not associated with the FEV_1_/FVC ratio (Table 4), and adjustment for individuals confounders or groups of confounders had little impact (Additional file 3: Table S3) (all *p*-values > 0.34). In sensitivity analyses, adjusting for laboratory batch had no impact on the interpretation of any the final models (data not shown).

**Table 3:** Adjusted mean levels of FVC by quartile of inflammatory biomarkers and associations per IQR change

CRP (mg/L)							
	Q1 (0.07–0.99)	Q2 (1.00–2.41)	Q3 (2.42–6.91)	Q4 (6.92–161.56)	p-for trend	β (95% CI) L FEV1 per 5.91 mg/L CRP	*p*-value
N	77	78	78	78	311	311	311
Basic^a^	4.02 (3.82, 4.23)	3.68 (3.47, 3.88)	3.42 (3.21, 3.62)	3.27 (3.07, 3.48)	<.0001	−82.09 (−126.69,-37.49)	0.0004
Fully adjusted	3.84 (3.63, 4.05)	3.65 (3.46, 3.83)	3.52 (3.33, 3.71)	3.39 (3.20, 3.59)	0.014	−65.50 (−106.61,-24.60)	0.0019
Parsimonious adjusted^b^	3.83 (3.64, 4.02)	3.63 (3.45, 3.82)	3.53 (3.34, 3.71)	3.40 (3.22, 3.59)	0.007	−60.22 (−99.61,-20.84)	0.003
IL-6 (pg/mL)							
	Q1 (0.30–1.26)	Q2 (1.27–2.12)	Q3 (2.13–4.44)	Q4 (4.45–46.8)	p-for trend	β (95% CI) mL FEV1 per 3.18 pg/mL IL-6	
N	77	83	76	75	311	311	311
Basic^a^	3.85 (3.63, 4.06)	3.69 (3.49, 3.89)	3.55 (3.33, 3.76)	3.29 (3.08, 3.50)	0.0004	−125.13 (−194.85,-55.40)	0.0005
Fully adjusted	3.73 (3.53, 3.93)	3.62 (3.44, 3.80)	3.61 (3.42, 3.81)	3.43 (3.23, 3.63)	0.0481	−77.69 (−143.93,-11.46)	0.0222
Parsimonious adjusted^b^	3.73 (3.54, 3.92)	3.62 (3.44, 3.80)	3.62 (3.43, 3.81)	3.42 (3.23, 3.61)	0.0307	−76.87 (−139.55,-14.19)	0.0169

^a^Adjusted for age, sex, race, and height

^b^Adjusted for age, sex, race, height, smoking status and pack-years, marijuana smoking status, COPD or asthma, current use of steroids and long-acting bronchodilators, level/severity of injury (LOI), and wheelchair use

**Table 4:** Adjusted mean levels of FEV1/FVC(%) by quartile of inflammatory biomarkers and associations per IQR change

CRP (mg/L)							
	Q1 (0.07–0.99)	Q2 (1.00–2.41)	Q3 (2.42–6.91)	Q4 (6.92–161.56)	p-for trend	β (95% CI) FEV1/FVC per 5.91 mg/L CRP	*p*-value
N	77	78	78	78	311	311	311
Basic^a^	76.0 (73.8, 78.2)	77.2 (75.0, 79.4)	76.2 (74.0, 78.4)	78.4 (76.2, 80.6)	0.14	−0.068 (−0.536,0.40)	0.78
Fully adjusted	76.4 (74.1, 78.7)	77.3 (75.2, 79.3)	76.3 (74.3, 78.4)	77.8 (75.6, 79.9)	0.43	−0.19 (−0.643,0.262)	0.41
Parsimonious adjusted^b^	76.8 (74.7, 79.0)	77.3 (75.3, 79.4)	76.0 (74.0, 78.1)	77.6 (75.5, 79.6)	0.59	−0.216 (−0.65,0.217)	0.33
IL-6 (pg/mL)							
	Q1 (0.30–1.26)	Q2 (1.27–2.12)	Q3 (2.13–4.44)	Q4 (4.45–46.8)	p-for trend	β (95% CI) FEV1/FVC per 3.18 pg/mL IL-6	
N	77	83	76	75	311	311	311
Basic^a^	77.6 (75.3, 79.8)	76.9 (74.7, 79.0)	77.1 (74.9, 79.4)	76.2 (74.0, 78.5)	0.47	−0.13 (−0.859,0.599)	0.73
Fully adjusted	77.5 (75.3, 79.7)	76.6 (74.6, 78.6)	77.5 (75.4, 79.6)	76.2 (74.0, 78.4)	0.50	0.069 (−0.654,0.791)	0.85
Parsimonious adjusted^b^	77.9 (75.8, 79.9)	76.5 (74.5, 78.4)	77.4 (75.3, 79.5)	76.1 (74.0, 78.2)	0.38	−0.041 (−0.726,0.645)	0.91

^a^Adjusted for age, sex, and race

^b^Adjusted for age, sex, race, smoking status and pack-years, marijuana smoking status, COPD or asthma, current use of steroids and long-acting bronchodilators, level/severity of injury (LOI), and wheelchair use

## Discussion

In this cohort of individuals with SCI, biomarkers of systemic inflammation (CRP, IL-6) were associated with decreases in FEV_1_ and FVC, but not FEV_1_/FVC. These findings were robust to adjustment for a number of potential confounders, including demographics and anthropometrics (age, race, sex, height, and BMI), lifestyle characteristics (cigarette and marijuana smoking status, medication use, and usual mobility mode), and disease characteristics (level of injury, history of COPD or asthma, history of chest operations or chest injuries).

In our previous analyses of 59 and 137 participants with SCI studied between 2003 and 2007, we observed similar associations between measures of inflammation and pulmonary function [13, 14]. In our pilot study of 59 individuals, IL-6 was inversely associated with percent-predicted FEV_1_ (mean percent-predicted FEV_1_ 92.4% in the least exposed quartile and 69.8% in the most exposed) and percent-predicted FVC (mean percent-predicted FVC 86.9% in the least exposed quartile and 71.5% in the most exposed) in unadjusted models, and in models adjusted for either SCI level, history of doctor diagnosed COPD, cigarette smoking status, or BMI (multivariable models were not possible). Similar decreases that did not reach statistical significance were observed between CRP and percent-predicted FEV_1_ or percent-predicted FVC, and no associations were observed with FEV_1_/FVC [13]. In a larger study of 137 individuals (54 of whom were also participants in the pilot study), we observed similar findings, even in multivariable models simultaneously adjusted for level of injury, BMI, cigarette smoking, statin use, and doctor-diagnosed COPD [14]. In our current study conducted in a larger SCI cohort enrolled between 2009 and 2015, we observed little confounding, and our parsimonious models included a different set of confounders than our previous studies (age, sex, race, height, cigarette smoking status and pack-years, marijuana smoking status, doctor diagnosed COPD, level or injury and mobility mode (dichotomized into wheelchair vs walking aided or unaided), and use of inhaled steroids or long-acting bronchodilators). Overall, we have observed consistent decreases in FEV_1_ and FVC with increases in CRP and IL-6 among populations of individuals with SCI.

Our findings are also consistent with most other cross-sectional studies [1–17]. Across a wide variety of populations, including individuals with and without chronic illnesses, increases in markers of systemic inflammation have been associated with declines in measures of pulmonary function. Similar to our study, the majority of the literature has focused on the impacts of CRP and IL-6 on FEV_1_ and FVC.

This study has a number of limitations. First, due to its cross-sectional nature, we cannot determine the temporality of the associations between increased inflammation and decreased pulmonary function. A recent longitudinal study among a group of younger adults studied at ages 32 and 38 has suggested that reductions in pulmonary function lead to subsequent increases in inflammation, but that inflammation did not predict future decreases in lung function [24]. This is contrary to another longitudinal study that found CRP measured in young adults was predictive of pulmonary function measured 7 years later, and other studies that have suggested inflammation may be related to subsequent pulmonary function [18–20].

Second, although we have considered an extensive number of potential confounders that were risk factors for pulmonary function and shown that our associations are robust to adjustment, residual confounding is always a concern in epidemiologic studies. Thirdly, the mechanism whereby systemic inflammation could influence pulmonary function in SCI is uncertain. Since chronic SCI is not known to be a condition characterized by pulmonary inflammation, it is likely that systemic inflammation following SCI is a function of decreased mobility, pressure ulcers, bladder dysfunction, and increased adipose tissue [32, 38]. Once these factors that occur after SCI are accounted for, we have previously found that level and completeness of SCI is not associated with CRP [32, 38]. However, since systemic inflammation is associated with muscle weakness and frailty, it is possible that systemic inflammation could adversely affect respiratory muscle performance and contribute to reduced pulmonary function [39–42].

Lastly, our study population may not be broadly generalizable. We have small numbers of female and minority participants, reflecting the distribution in the population served at VA Boston. However, associations between inflammation and pulmonary function have been observed across a wide spectrum of populations.

## Conclusions

Plasma CRP and IL-6 in individuals with chronic SCI is inversely associated with FEV_1_ and FVC, independent of SCI severity, BMI, and other covariates. This finding suggests that systemic inflammation may contribute to reduced pulmonary function in chronic SCI.

## Additional files

Additional file 1: Table S1. Univariate adjusted mean levels of FEV1 by quartile of inflammatory biomarkers and associations per IQR change. (DOCX 48 kb)

Additional file 2: Table S2. Univariate Adjusted mean levels of FVC by quartile of inflammatory biomarkers and associations per IQR change. (DOCX 49 kb)

Additional file 3: Table S3. Univariate adjusted mean levels of FEV1/FVC(%) by quartile of inflammatory biomarkers and associations per IQR change. (DOCX 48 kb)

## Abbreviations

AIS: American spinal injury association impairment scale
ATS: American Thoracic Society
BMI: Body mass index
CI: Confidence interval
COPD: Chronic obstructive pulmonary disease
CRP: C-reactive protein
FEV1: Forced expiratory volume in 1 s
FVC: Forced vital capacity
IL-6: Interleukin-6
IQR: Interquartile range
NSCIA: National Spinal Cord Injury Association
RV: Residual volume
SCI: Spinal cord injury
VA: Veteran Affairs
