# Supplementary material for: FEV1 and FVC and systemic inflammation in a spinal cord injury cohort
Source: BMC Pulm Med. 2017 Aug 15;17:113. doi: 10.1186/s12890-017-0459-6 (PMC5558736; doi:10.1186/s12890-017-0459-6)
Supplement: Supplementary file 1 — Univariate adjusted mean levels of FEV1 by quartile of inflammatory biomarkers and associations per IQR change. (DOCX 48 kb) [file 12890_2017_459_MOESM1_ESM.docx]

| **Additional file 1 Table S1: Univariate adjusted mean levels of FEV1 by quartile of inflammatory biomarkers and associations per IQR change** | | | | | | | |
| --- | --- | --- | --- | --- | --- | --- | --- |
|  | **CRP (mg/L)** | | | | | | |
|  | **Q1**  **(0.07-0.99)** | **Q2**  **(1.00-2.41)** | **Q3**  **(2.42-6.91)** | **Q4**  **(6.92-161.56)** | **p-for trend** | **β (95% CI) mL FEV1 per 5.91 mg/L CRP** | **p-value** |
| N | 77 | 78 | 78 | 78 | 311 | 311 | 311 |
| Basic + BMI | 3.08 (2.91, 3.26) | 2.84 (2.68, 3.01) | 2.58 (2.41, 2.74) | 2.53 (2.36, 2.70) | 0.0003 | -63.18 (-99.09,-27.27) | 0.0007 |
| Basic + statins | 3.05 (2.89, 3.22) | 2.84 (2.68, 3.01) | 2.59 (2.43, 2.75) | 2.55 (2.38, 2.71) | 0.0002 | -65.07 (-100.98,-29.16) | 0.0004 |
| Basic + BDs + steroids | 3.05 (2.89, 3.22) | 2.83 (2.67, 3.00) | 2.59 (2.43, 2.76) | 2.55 (2.39, 2.72) | 0.0003 | -64.12 (-99.69,-28.56) | 0.0005 |
| Basic + LOI | 2.96 (2.80, 3.12) | 2.81 (2.65, 2.96) | 2.64 (2.49, 2.80) | 2.62 (2.46, 2.78) | 0.0139 | -50.77 (-83.66,-17.87) | 0.0027 |
| Basic + mobility mode | 2.90 (2.74, 3.06) | 2.81 (2.66, 2.97) | 2.65 (2.50, 2.81) | 2.66 (2.50, 2.82) | 0.0840 | -40.13 (-73.26,-7.00) | 0.0182 |
| Basic + LOI + mobility mode | 2.96 (2.80, 3.12) | 2.81 (2.65, 2.96) | 2.64 (2.49, 2.80) | 2.62 (2.47, 2.78) | 0.0159 | -50.35 (-83.25,-17.46) | 0.003 |
| Basic + COPD or asthma | 3.06 (2.90, 3.22) | 2.84 (2.68, 3.00) | 2.60 (2.43, 2.76) | 2.54 (2.37, 2.70) | <.0001 | -68.85 (-103.72,-33.98) | 0.0001 |
| Basic + chest injury | 3.07 (2.90, 3.23) | 2.85 (2.68, 3.01) | 2.58 (2.42, 2.75) | 2.54 (2.37, 2.70) | 0.0001 | -64.83 (-100.39,-29.27) | 0.0004 |
| Basic + smoking | 3.04 (2.88, 3.21) | 2.84 (2.68, 3.01) | 2.60 (2.44, 2.76) | 2.55 (2.38, 2.71) | 0.0002 | -63.24 (-98.80,-27.68) | 0.0006 |
| Basic +marijuana | 3.04 (2.88, 3.21) | 2.85 (2.69, 3.02) | 2.59 (2.42, 2.75) | 2.55 (2.39, 2.72) | 0.0003 | -62.11 (-97.68,-26.55) | 0.0007 |
|  | **IL-6 (pg/mL)** | | | | | | |
|  | **Q1**  **(0.30-1.26)** | **Q2**  **(1.27-2.12)** | **Q3**  **(2.13-4.44)** | **Q4**  **(4.45-46.8)** | **p-for trend** | **β (95% CI) mL FEV1 per 3.18 pg/mL IL-6** | **p-value** |
| N | 77 | 83 | 76 | 75 | 311 | 311 | 311 |
| Basic + BMI | 3.00 (2.82, 3.17) | 2.82 (2.66, 2.98) | 2.71 (2.54, 2.87) | 2.49 (2.32, 2.67) | 0.0004 | -103.29 (-160.92,-45.65) | 0.0005 |
| Basic + statins | 2.98 (2.81, 3.15) | 2.82 (2.66, 2.98) | 2.71 (2.54, 2.88) | 2.51 (2.34, 2.68) | 0.0003 | -101.28 (-156.86,-45.70) | 0.0004 |
| Basic + BDs + steroids | 2.99 (2.82, 3.16) | 2.82 (2.66, 2.98) | 2.71 (2.54, 2.88) | 2.51 (2.34, 2.68) | 0.0003 | -102.05 (-157.62,-46.47) | 0.0004 |
| Basic + LOI | 2.93 (2.77, 3.09) | 2.78 (2.63, 2.93) | 2.72 (2.57, 2.88) | 2.58 (2.43, 2.74) | 0.0064 | -78.36 (-129.77,-26.96) | 0.0030 |
| Basic + mobility mode | 2.87 (2.71, 3.03) | 2.78 (2.63, 2.93) | 2.80 (2.65, 2.96) | 2.57 (2.41, 2.73) | 0.0076 | -60.97 (-112.81,-9.13) | 0.0218 |
| Basic + LOI + mobility mode | 2.93 (2.77, 3.09) | 2.78 (2.63, 2.93) | 2.72 (2.57, 2.88) | 2.59 (2.43, 2.75) | 0.0080 | -77.41 (-128.94,-25.88) | 0.0035 |
| Basic + COPD or asthma | 2.98 (2.82, 3.15) | 2.80 (2.64, 2.96) | 2.72 (2.55, 2.88) | 2.52 (2.35, 2.68) | 0.0004 | -95.24 (-150.07,-40.41) | 0.0007 |
| Basic + chest injury | 3.00 (2.83, 3.17) | 2.81 (2.65, 2.97) | 2.70 (2.53, 2.87) | 2.50 (2.33, 2.67) | 0.0002 | -102.78 (-158.29,-47.26) | 0.0003 |
| Basic + smoking | 2.98 (2.81, 3.15) | 2.81 (2.65, 2.98) | 2.72 (2.55, 2.89) | 2.50 (2.33, 2.67) | 0.0003 | -100.36 (-156.31,-44.41) | 0.0005 |
| Basic + marijuana | 2.98 (2.81, 3.15) | 2.81 (2.65, 2.97) | 2.71 (2.55, 2.88) | 2.52 (2.35, 2.69) | 0.0005 | -96.99 (-152.69,-41.29) | 0.0007 |
