# Supplementary material for: FEV1 and FVC and systemic inflammation in a spinal cord injury cohort
Source: BMC Pulm Med. 2017 Aug 15;17:113. doi: 10.1186/s12890-017-0459-6 (PMC5558736; doi:10.1186/s12890-017-0459-6)
Supplement: Supplementary file 2 — Univariate Adjusted mean levels of FVC by quartile of inflammatory biomarkers and associations per IQR change. (DOCX 49 kb) [file 12890_2017_459_MOESM2_ESM.docx]

| **Additional file 2: Table S2. Univariate Adjusted mean levels of FVC by quartile of inflammatory biomarkers and associations per IQR change** | | | | | | | |
| --- | --- | --- | --- | --- | --- | --- | --- |
|  | **CRP (mg/L)** | | | | | | |
|  | **Q1**  **(0.07-0.99)** | **Q2**  **(1.00-2.41)** | **Q3**  **(2.42-6.91)** | **Q4**  **(6.92-161.56)** | **p-for trend** | **β (95% CI) L FEV1 per 5.91 mg/L CRP** | **p-value** |
| N | 77 | 78 | 78 | 78 | 311 | 311 | 311 |
| Basic + BMI | 4.08 (3.87, 4.3) | 3.69 (3.48, 3.89) | 3.40 (3.19, 3.60) | 3.23 (3.02, 3.44) | <.0001 | -82.74 (-127.80,-37.68) | 0.0004 |
| Basic + statins | 4.03 (3.82, 4.23) | 3.68 (3.48, 3.89) | 3.42 (3.22, 3.62) | 3.27 (3.06, 3.47) | <.0001 | -83.39 (-128.45,-38.33) | 0.0003 |
| Basic + BDs + steroids | 4.03 (3.82, 4.23) | 3.68 (3.48, 3.89) | 3.42 (3.21, 3.62) | 3.27 (3.07, 3.47) | <.0001 | -82.21 (-126.92,-37.50) | 0.0004 |
| Basic + LOI | 3.87 (3.68, 4.06) | 3.62 (3.44, 3.81) | 3.50 (3.31, 3.68) | 3.40 (3.21, 3.58) | 0.005 | -61.29 (-100.79,-21.79) | 0.0026 |
| Basic + mobility mode | 3.78 (3.59, 3.98) | 3.64 (3.46, 3.83) | 3.52 (3.33, 3.70) | 3.45 (3.26, 3.64) | 0.042 | -46.45 (-86.53,-6.37) | 0.0237 |
| Basic + LOI + mobility mode | 3.85 (3.66, 4.04) | 3.63 (3.44, 3.81) | 3.51 (3.32, 3.69) | 3.40 (3.21, 3.59) | 0.006 | -59.99 (-99.37,-20.60) | 0.0031 |
| Basic + COPD or asthma | 4.03 (3.82, 4.23) | 3.68 (3.48, 3.89) | 3.42 (3.22, 3.62) | 3.26 (3.06, 3.47) | <.0001 | -84.34 (-129.05,-39.62) | 0.0003 |
| Basic + chest injury | 4.04 (3.83, 4.25) | 3.69 (3.49, 3.90) | 3.41 (3.21, 3.61) | 3.25 (3.05, 3.46) | <.0001 | -83.63 (-128.22,-39.03) | 0.0003 |
| Basic + smoking | 4.02 (3.82, 4.23) | 3.67 (3.46, 3.87) | 3.44 (3.23, 3.64) | 3.27 (3.06, 3.47) | <.0001 | -83.51 (-128.11,-38.91) | 0.0003 |
| Basic +marijuana | 4.01 (3.80, 4.22) | 3.69 (3.49, 3.90) | 3.41 (3.21, 3.61) | 3.28 (3.08, 3.48) | <.0001 | -78.90 (-123.26,-34.53) | 0.0006 |
|  | **IL-6 (pg/mL)** | | | | | | |
|  | **Q1**  **(0.30-1.26)** | **Q2**  **(1.27-2.12)** | **Q3**  **(2.13-4.44)** | **Q4**  **(4.45-46.8)** | **p-for trend** | **β (95% CI) mL FEV1 per 3.18 pg/mL IL-6** |  |
| N | 77 | 83 | 76 | 75 | 311 | 311 | 311 |
| Basic + BMI | 3.87 (3.65, 4.09) | 3.70 (3.49, 3.90) | 3.54 (3.33, 3.76) | 3.26 (3.04, 3.48) | 0.0003 | -131.48 (-203.89,-59.08) | 0.0004 |
| Basic + statins | 3.85 (3.63, 4.06) | 3.69 (3.49, 3.89) | 3.55 (3.33, 3.76) | 3.29 (3.07, 3.50) | 0.0004 | -125.22 (-195.07,-55.37) | 0.0005 |
| Basic + BDs + steroids | 3.86 (3.64, 4.07) | 3.70 (3.50, 3.90) | 3.54 (3.32, 3.75) | 3.28 (3.07, 3.50) | 0.0003 | -126.59 (-196.56,-56.62) | 0.0005 |
| Basic + LOI | 3.75 (3.56, 3.94) | 3.64 (3.46, 3.82) | 3.59 (3.40, 3.78) | 3.40 (3.21, 3.59) | 0.0141 | -88.12 (-149.99,-26.25) | 0.0056 |
| Basic + mobility mode | 3.66 (3.47, 3.85) | 3.64 (3.46, 3.82) | 3.69 (3.50, 3.88) | 3.39 (3.20, 3.58) | 0.0217 | -66.03 (-128.77,-3.28) | 0.0400 |
| Basic + LOI + mobility mode | 3.74 (3.55, 3.93) | 3.63 (3.46, 3.81) | 3.59 (3.40, 3.78) | 3.41 (3.22, 3.60) | 0.0217 | -85.23 (-147.04,-23.42) | 0.0072 |
| Basic + COPD or asthma | 3.85 (3.63, 4.06) | 3.69 (3.48, 3.89) | 3.55 (3.34, 3.76) | 3.29 (3.08, 3.51) | 0.0005 | -123.25 (-193.16,-53.34) | 0.0006 |
| Basic + chest injury | 3.87 (3.65, 4.08) | 3.69 (3.48, 3.89) | 3.54 (3.33, 3.75) | 3.28 (3.07, 3.50) | 0.0003 | -127.16 (-196.88,-57.44) | 0.0004 |
| Basic + smoking | 3.86 (3.64, 4.07) | 3.70 (3.50, 3.90) | 3.55 (3.33, 3.76) | 3.27 (3.05, 3.48) | 0.0002 | -133.04 (-203.26,-62.82) | 0.0002 |
| Basic + marijuana | 3.83 (3.62, 4.05) | 3.68 (3.48, 3.88) | 3.55 (3.34, 3.77) | 3.31 (3.09, 3.52) | 0.0009 | -117.34 (-187.12,-47.55) | 0.0011 |
