# Supplementary material for: FEV1 and FVC and systemic inflammation in a spinal cord injury cohort
Source: BMC Pulm Med. 2017 Aug 15;17:113. doi: 10.1186/s12890-017-0459-6 (PMC5558736; doi:10.1186/s12890-017-0459-6)
Supplement: Supplementary file 3 — Univariate adjusted mean levels of FEV1/FVC(%) by quartile of inflammatory biomarkers and associations per IQR change. (DOCX 48 kb) [file 12890_2017_459_MOESM3_ESM.docx]

| **Additional file 3 Table S3: Univariate adjusted mean levels of FEV1/FVC(%) by quartile of inflammatory biomarkers and associations per IQR change** | | | | | | | |
| --- | --- | --- | --- | --- | --- | --- | --- |
|  | **CRP (mg/L)** | | | | | | |
|  | **Q1**  **(0.07-0.99)** | **Q2**  **(1.00-2.41)** | **Q3**  **(2.42-6.91)** | **Q4**  **(6.92-161.56)** | **p-for trend** | **β (95% CI) FEV1/FVC per 5.91 mg/L CRP** | **p-value** |
| N | 77 | 78 | 78 | 78 | 311 | 311 | 311 |
| Basic + BMI | 75.3 (73.0, 77.6) | 77.1 (74.9, 79.3) | 76.4 (74.2, 78.6) | 78.9 (76.7, 81.1) | 0.05 | -0.016 (-0.488,0.457) | 0.95 |
| Basic + statins | 76.0 (73.8, 78.2) | 77.2 (75.0, 79.4) | 76.2 (74.0, 78.4) | 78.4 (76.2, 80.6) | 0.16 | -0.088 (-0.561,0.384) | 0.71 |
| Basic + BDs + steroids | 76.0 (73.8, 78.2) | 77.0 (74.8, 79.1) | 76.3 (74.1, 78.5) | 78.5 (76.4, 80.7) | 0.11 | -0.077 (-0.538,0.385) | 0.74 |
| Basic + LOI | 76.9 (74.7, 79.1) | 77.5 (75.3, 79.6) | 75.9 (73.7, 78.0) | 77.6 (75.4, 79.8) | 0.62 | -0.162 (-0.621,0.298) | 0.49 |
| Basic + mobility mode | 77.2 (74.9, 79.4) | 77.3 (75.2, 79.5) | 75.7 (73.6, 77.9) | 77.6 (75.4, 79.8) | 0.66 | -0.198 (-0.662,0.266) | 0.40 |
| Basic + LOI + mobility mode | 77.2 (75.0, 79.4) | 77.3 (75.2, 79.5) | 75.7 (73.6, 77.8) | 77.5 (75.4, 79.7) | 0.71 | -0.179 (-0.636,0.278) | 0.44 |
| Basic + COPD or asthma | 76.2 (74.1, 78.3) | 77.2 (75.1, 79.3) | 76.3 (74.2, 78.4) | 78.1 (76.0, 80.2) | 0.22 | -0.167 (-0.612,0.277) | 0.46 |
| Basic + chest injury | 76.0 (73.7, 78.2) | 77.2 (75.0, 79.4) | 76.2 (74.0, 78.4) | 78.5 (76.3, 80.7) | 0.13 | -0.066 (-0.535,0.403) | 0.78 |
| Basic + smoking | 75.7 (73.6, 77.9) | 77.5 (75.4, 79.7) | 76.1 (74.0, 78.3) | 78.4 (76.3, 80.5) | 0.13 | -0.021 (-0.477,0.436) | 0.93 |
| Basic +marijuana | 76.0 (73.8, 78.2) | 77.2 (75.0, 79.4) | 76.3 (74.1, 78.5) | 78.3 (76.1, 80.5) | 0.16 | -0.092 (-0.561,0.376) | 0.70 |
|  | **IL-6 (pg/mL)** | | | | | | |
|  | **Q1**  **(0.30-1.26)** | **Q2**  **(1.27-2.12)** | **Q3**  **(2.13-4.44)** | **Q4**  **(4.45-46.8)** | **p-for trend** | **β (95% CI) FEV1/FVC per 3.18 pg/mL IL-6** |  |
| N | 77 | 83 | 76 | 75 | 311 | 311 | 311 |
| Basic + BMI | 77.2 (74.9, 79.5) | 76.8 (74.7, 78.9) | 77.2 (74.9, 79.4) | 76.6 (74.3, 79.0) | 0.79 | 0.024 (-0.73,0.778) | 0.95 |
| Basic + statins | 77.6 (75.3, 79.8) | 76.9 (74.7, 79.0) | 77.1 (74.9, 79.3) | 76.3 (74.0, 78.5) | 0.48 | -0.125 (-0.854,0.604) | 0.74 |
| Basic + BDs + steroids | 77.4 (75.2, 79.6) | 76.7 (74.6, 78.8) | 77.3 (75.1, 79.6) | 76.4 (74.2, 78.6) | 0.59 | -0.125 (-0.842,0.591) | 0.73 |
| Basic + LOI | 78.2 (76.0, 80.4) | 77.0 (75.0, 79.1) | 76.7 (74.5, 78.9) | 75.8 (73.6, 78.0) | 0.18 | -0.307 (-1.024,0.41) | 0.40 |
| Basic + mobility mode | 78.4 (76.2, 80.7) | 77.0 (75.0, 79.1) | 76.6 (74.4, 78.8) | 75.7 (73.5, 77.9) | 0.15 | -0.315 (-1.038,0.407) | 0.39 |
| Basic + LOI + mobility mode | 78.3 (76.2, 80.5) | 77.1 (75.0, 79.2) | 76.7 (74.5, 78.9) | 75.6 (73.4, 77.8) | 0.12 | -0.347 (-1.057,0.364) | 0.34 |
| Basic + COPD or asthma | 77.5 (75.4, 79.7) | 76.5 (74.5, 78.5) | 77.4 (75.3, 79.5) | 76.4 (74.3, 78.5) | 0.59 | 0.031 (-0.661,0.722) | 0.93 |
| Basic + chest injury | 77.5 (75.3, 79.8) | 76.9 (74.7, 79.0) | 77.1 (74.9, 79.4) | 76.3 (74.0, 78.5) | 0.48 | -0.127 (-0.856,0.602) | 0.73 |
| Basic + smoking | 77.2 (75.0, 79.4) | 76.5 (74.4, 78.6) | 77.5 (75.3, 79.7) | 76.6 (74.4, 78.8) | 0.77 | 0.086 (-0.631,0.802) | 0.81 |
| Basic + marijuana | 77.7 (75.4, 79.9) | 76.9 (74.7, 79.0) | 77.1 (75.0, 79.4) | 76.1 (73.8, 78.3) | 0.38 | -0.183 (-0.912,0.546) | 0.62 |
